# Validity and reproducibility of retinal arteriole and venule diameter measurements: ELSA-Brasil study. A cross-sectional study

**DOI:** 10.1590/1516-3180.2018.0227230718

**Published:** 2018-07-21

**Authors:** William Jones Dartora, Marcelo Krieger Maestri, Maria Inês Schmidt, Bruce Bartholow Duncan, Lloyd Chambless, Ronald Klein, Stacy Meuer, Vivian Cristine Luft

**Affiliations:** I MSc. Doctoral Student, Postgraduate Program on Epidemiology, Universidade Federal do Rio Grande do Sul (UFRGS), Porto Alegre (RS), Brazil.; II MD, PhD. Adjunct Professor, Hospital de Clínicas de Porto Alegre, Universidade Federal do Rio Grande do Sul (UFRGS), Porto Alegre (RS), Brazil.; III MD, PhD. Full Professor, Postgraduate Program on Epidemiology, Universidade Federal do Rio Grande do Sul (UFRGS), Porto Alegre (RS) Brazil.; IV MD, PhD. Full Professor, Postgraduate Program on Epidemiology, Universidade Federal do Rio Grande do Sul (UFRGS), Porto Alegre (RS), Brazil.; V PhD. Research Professor, Department of Biostatistics, University of North Carolina, Chapel Hill, North Carolina, United States.; VI MD, MPH. Professor, Department of Ophthalmology and Visual Sciences, University of Wisconsin, Madison, United States.; VII BS. Research, Department of Ophthalmology and Visual Sciences, University of Wisconsin, Madison, United States.; VIII PhD. Adjunct Professor, Postgraduate Program on Epidemiology, Universidade Federal do Rio Grande do Sul (UFRGS), Porto Alegre (RS), Brazil.

**Keywords:** Microvessels, Retinal artery, Retinal vein, Reproducibility of results

## Abstract

**BACKGROUND:**

Investigation of alterations to retinal microvasculature may contribute towards understanding the role of such changes in the pathophysiology of several chronic non-communicable diseases. The objective here was to evaluate the validity and reproducibility of retinal arteriole and venule diameter measurements made by Brazilian Longitudinal Study of Adult Health (ELSA-Brasil) graders.

**DESIGN AND SETTING:**

Cross-sectional study at six teaching and research institutions.

**METHODS:**

To evaluate validity, each of 25 retinal images from the University of Wisconsin (gold standard) was measured by five ELSA-Brasil graders. To evaluate reproducibility, 105 images across the spectrum of vessel diameters were selected from 12,257 retinal images that had been obtained between 2010 and 2012, and each image was reexamined by the same grader and by an independent grader. All measurements were made using the Interactive Vessel Analysis (IVAN) software. Bland-Altman plots, paired t tests and intraclass correlation coefficients (ICCs) were analyzed.

**RESULTS:**

Mean differences between ELSA-Brasil and gold-standard readings were 0.16 µm (95% CI -0.17-0.50; P = 0.31) for central retinal artery equivalent (CRAE), -0.21 µm (95% CI -0.56-0.14; P = 0.22) for central retinal vein equivalent (CRVE) and 0.0005 (95% CI -0.008-0.009; P = 0.55) for arteriole/venule ratio (AVR). Intragrader ICCs were 0.77 (95% CI 0.67-0.86) for CRAE, 0.90 (95% CI 0.780.96) for CRVE and 0.70 (0.55-0.83) for AVR. Intergrader ICCs were 0.75 (95% CI 0.64-0.85) for CRAE, 0.90 (95% CI 0.79-0.96) for CRVE and 0.68 (95% CI 0.55-0.82) for AVR.

**CONCLUSIONS:**

Retinal microvascular diameter measurements are valid and present moderate to high intra and intergrader reproducibility in ELSA-Brasil.

## INTRODUCTION

Metabolic and vascular changes are inherent to the pathophysiology of diabetes and cardiovascular diseases, and their complications may cause damage to the microvasculature. This is reflected in changes to the diameters of retinal microvessels.^1^ ^2^^,^^3^^,^^4^^,^^5^^,^^6^^,^^7^^,^^8^^,^^9^^,^^10^ In this regard, the microvasculature of the retina is considered to be a non-invasive window to the microvascular system, which thus allows inferences to be made in relation to its involvement in the etiology of chronic diseases.^11^ Furthermore, changes to retinal vessel caliber have been correlated with incident hypertension, diabetes and cerebral vascular disease.^12^^,^^13^^,^^14^

To be useful, microvascular measurements of the retina need to be accurate and precise. The approaches towards making such measurements on the retina are still not well described in the literature, most probably because some studies have not defined well which tools (­software and mathematical calculations) are used to measure these data. In this regard, the aim of the present study was to evaluate the validity and reproducibility of retinal microvascular measurements that were made within the Brazilian Longitudinal Study of Adult Health (ELSA-Brasil).

## METHODS

The Brazilian Longitudinal Study of Adult Health (ELSA-Brasil) is a multicenter study on 15,105 volunteer staff members (aged 35-74 years) at public institutions of higher education in six Brazilian state capital cities who were enrolled between 2008 and 2012. The overall objective of ELSA-Brasil was to investigate the epidemiological, clinical and molecular aspects of non-communicable chronic diseases, especially cardiovascular diseases and diabetes. ELSA Brasil was approved by the Ethics Committees of Hospital de Clínicas de Porto Alegre (under the registration number 06-194), Hospital Universitário da Universidade de São Paulo (669/06), Fundação Oswaldo Cruz (343/06), Universidade Federal de Minas Gerais (186/06), Universidade Federal da Bahia (027-06) and Universidade Federal do Espírito Santo (041/06), and all participants consented to their participation in the research.^15^^,^^16^

Sociodemographic and health data from the ELSA participants were collected through interviews and examinations, as previously described in greater detail.^17^ Retinal images were obtained using a Canon CR-1 digital non-mydriatic retinal camera, coupled to a Canon EOS 40D digital camera (10 MP), and the images were shot at a 45º angle. Photographs of the macula and optic disc were acquired from both eyes of each participant. The retinal images were obtained in accordance with pre-established protocols,^18^ without compression or zooming of images, so as not to lose quality.^1^^,^^19^^,^^20^ The images were then transferred in Digital Imaging and Communications in Medicine (DICOM) format to the study’s central server, from which they were retrieved by the reading center. A small fraction of the images were stored in .jpeg format, without compression, for transfer.

The supervisory ophthalmologist of the ELSA-Brasil retinal reading center was trained and certified by professionals in the Ocular Epidemiology Research Group of the Department of Ophthalmology and Visual Sciences, University of Wisconsin-Madison (UW), to make microvascular retinal measurements using the Interactive Vessel Analysis (IVAN) software.^20^ He then trained and supervised the graders who performed the analyses in ELSA-Brasil, who were healthcare professionals without prior knowledge of ocular fundus images. The five readers who performed the greatest numbers of readings (28% of the total) were chosen to participate in the evaluations of validity and reproducibility.

For the validity evaluation, 25 retinal images received from the Ocular Epidemiology Research Group (UW) that had previously been read were considered to be the gold standard for ELSA-Brasil readings. Five evaluators from the ELSA-Brasil study read each of the 25 images received, totaling 125 readings.

For the reproducibility evaluation, images from ELSA-Brasil participants that had previously been read by these five graders were selected for new readings. In order to select images representing the range of values obtained, the images were classified into quintiles of their values, separately for central retinal artery equivalent (CRAE), central retinal vein equivalent (CRVE) and arterio-venous ratio (AVR, i.e. CRAE/CRVE). Seven images were selected from each quintile of each measurement for the study, thus totaling 105 images, of which 21 had been originally measured by each of the five graders.

Since a high correlation of retinal vessel caliber between the right and left eyes (0.78 to 0.99 intra and intergrader) had previously been shown,^21^ optical disc images of a single eye were used. The right eye was given preference for this. In this selection, images that did not open in the software, or which did not meet the minimum criteria for grading (low light, poor visual quality or less than four venules and/or arterioles present) were excluded.

As mentioned earlier, to assess reproducibility, each of the five graders re-read each of their 21 selected images, thus totaling 105 repeat readings. To evaluate intergrader reproducibility, each image was re-read by another grader, again totaling 105 repeated readings. Thus, considering both the original readings and the intra and intergrader repeated readings, the reproducibility evaluation was based on 315 readings (105 original readings + 105 readings repeated by the same reader + 105 readings repeated by another reader). The third readings were performed on average two years after the first readings (on the same images). The readers on this occasion were blinded with regard to the identity of the previous reader and to the values previously obtained.

### Statistical analysis

Shapiro-Wilk and Kolmogorov-Smirnov tests were used to evaluate the normality of the data. Differences between the readings were evaluated through Bland-Altman plots and were tested using the t test for paired samples.

The intraclass correlation coefficient (ICC) was used to evaluate reproducibility. Since each image was read twice by one grader and once by another grader, we used a two-factor random effects model (not nested), with estimation of variance components by means of the method of moments,^22^ using an in-house SAS 9.4 routine (SAS Institute, Inc., Cary, North Carolina, USA) that had been developed for this purpose (supplementary material). The ICCs were calculated from the variance components in the usual manner: overall grading ICC (for repeated readings with different graders) = (1 - [(between-grader variance + within-grader variance)/(total variance)]); and within-grader ICC = (1 - [(within-grader variance)/(total variance)]). Ninety-five percent confidence intervals were calculated by means of bootstrapping, with 2000 bootstrap samples of the images.^23^ Reproducibility was classified as proposed by Hinkle and Wiersma, as moderate (ICC 0.50 to 0.70), high (ICC 0.70 to 0.90) or very high (ICC 0.90 to 1.00).^24^

Statistical analyses were performed using the SAS statistical software, version 9.4 (for ICC calculations) and the Statistical Package for the Social Sciences (SPSS), version 18 (for creation of Bland-Altman plots).

## RESULTS

### Validity

The retinal microvascular measurements of the sample of 25 images provided by the University of Wisconsin Ocular Epidemiology Research Group were normally distributed, as presented in Table 1. The mean CRAEs measured by the ELSA-Brasil and the UW graders were 138.2 ± 11.6 µm and 138.0 ± 11.5 µm, respectively, with a mean difference of 0.16 µm (95% CI -0.17 to 0.50; P = 0.31). The mean CRVEs were 198.3 ± 21.7 µm and 198.5 ± 21.7 µm, respectively, resulting in a mean difference of -0.21 µm (95% CI -0.56 to 0.14; P = 0.22). The mean AVRs were both 0.70 ± 0.07, with a mean difference of 0.0005 µm (95% CI -0.008 to 0.009; P = 0.55).

**Table 1:** Comparison of microvascular measurements of the retina in the Brazilian Longitudinal Study of Adult Health (ELSA-Brasil) and at the University of Wisconsin Department of Ophthalmology (gold standard), n = 25

	ELSA-Brasil	Gold standard	Difference	P
	Mean (SE)	Mean (SE)	Mean (95% CI)	
Central retinal artery equivalent (µm)	138.2 (2.33)	138.0 (2.29)	0.16 (-0.17-0.50)	0.31
Central retinal vein equivalent (µm)	198.3 (4.34)	198.5 (4.33)	-0.21 (-0.56-0.14)	0.22
Arteriole/venule ratio	0.70 (0.013)	0.70 (0.013)	0.0005 (-0.008-0.009)	0.55

P-value of the t test for paired data. SE = standard error; CI = confidence interval.

**Figure 1:**
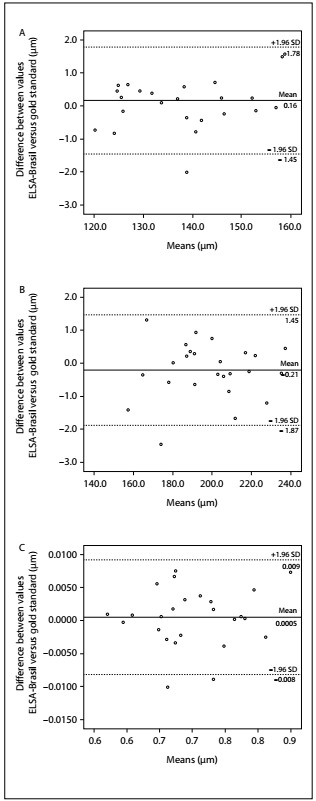
Validity evaluation on Bland-Altman plots of retinal microvascular measurements, comparing Brazilian Longitudinal Study of Adult Health (ELSA-Brasil) graders with those of the University of Wisconsin. Dashed lines indicate the 95% confidence interval (CI) of the mean difference, which is indicated by the black line.

The Bland-Altman graph (Figure 1) showed that the differences obtained between the means of the microvascular readings performed by the five ELSA-Brasil graders, in comparison with the Wisconsin readers, were distributed in a similar way over the spectrum of values of these measurements. The maximum value for the difference was 1.78 µm for the arteriole diameter (CRAE) and, in 84% of the images, the differences did not reach 1.0 µm. Venule measurements (CRVE) showed a maximum difference of 1.87 µm and 80% of the images had differences smaller than 1.0 µm. For AVRs, the maximum difference was 0.009 and, for 76% of the images, the differences were smaller than 0.005.

### Reproducibility

Among the 105 images selected, 63.8% were from women, 44.8% from whites, 48.6% from participants aged 45-54 years and 61.0% from those who had completed a university degree; 25.7% were from individuals with hypertension and 20.0% from individuals with diabetes (Table 2).

**Table 2:** Sociodemographic, anthropometric and disease characteristics of the Brazilian Longitudinal Study of Adult Health (ELSA-Brasil) sample that was used to evaluate microvascular measurement reproducibility, n = 105

Characteristic	N (%) or mean (standard deviation)	
Sex		
Female	67	(63.8)
Skin color/race		
White	47	(44.8)
Brown	33	(31.4)
Black	20	(19.1)
Others	5	(4.8)
Age (years)	51.4	(7.9)
Age strata		
35-44	22	(21.0)
45-54	51	(48.6)
55-64	24	(22.9)
65-74	8	(7.6)
Educational level		
Elementary school incomplete	2	(1.9)
Completed primary education	4	(3.8)
Completed high school	35	(33.3)
Completed college/university	64	(61.0)
Hypertension		
Yes	27	(25.7)
Diabetes mellitus		
Yes	21	(20.0)
Weight (kg)	72.5	(13.6)
Height (cm)	165.6	(9.7)
Body mass index (kg/m²)		
Men	26.3	( 4.1)
Women	26.3	(4.3)
Systolic blood pressure (mmHg)	117.9	(16.5)
Diastolic blood pressure (mmHg)	74.9	(10.7)

The distribution of retinal microvascular values in the reproducibility sample (n = 105) approximated the values found overall in ELSA-Brasil (n = 12,257), as shown in Table 3. Additionally, although the average differences between the original and repeat readings were at times statistically significant, they were always less than 3%, as follows: -2.55 µm (P = 0.02) for CRAE; 0.65 µm (P = 0.54) for CRVE; and -0.0015 µm (P = 0.02) for AVR.

**Table 3:** Distribution of retinal microvascular measurements in the sample that was used to calculate reproducibility (n = 105) and in the whole cohort of the Brazilian Longitudinal Study of Adult Health (ELSA-Brasil) (n = 12,257)

Variables	Sample mean (standard deviation)	Overall mean (standard deviation)
Central retinal artery equivalent (µm)	144.6 (15.5)	146.9 (15.0)
Central retinal vein equivalent (µm)	219.4 (24.7)	218.3 (20.6)
Arteriole/venule ratio	0.65 (0.08)	0.68 (0.07)

In the reproducibility evaluation, as seen in Table 4, the intragrader ICCs for CRAE, CRVE and AVR were 0.77 (95% CI 0.67 to 0.86), 0.90 (95% CI 0.78 to 0.96) and 0.70 (95% CI 0.55 to 0.83), respectively, and the intergrader ICCs were 0.75 (95% CI 0.64 to 0.85), 0.90 (95% CI 0.79 to 0.96) and 0.68 (95% CI 0.55-0.82), respectively.

**Table 4:** Intragrader and intergrader reproducibility of microvascular measurements of the retina in the Brazilian Longitudinal Study of Adult Health (ELSA-Brasil), n = 105

Measurement	Intragrader	Intergrader
	ICC (95% CI)	ICC (95% CI)
CRAE	0.77 (0.67-0.86)	0.75 (0.64-0.85)
CRVE	0.90 (0.78-0.96)	0.90 (0.79-0.96)
AVR	0.70 (0.55-0.83)	0.68 (0.55-0.82)

ICC = intraclass correlation coefficients; CI = confidence interval; CRAE = central retinal artery equivalent, CRVE = central retinal vein equivalent, AVR = arteriole/venule ratio.

**Figure 2:**
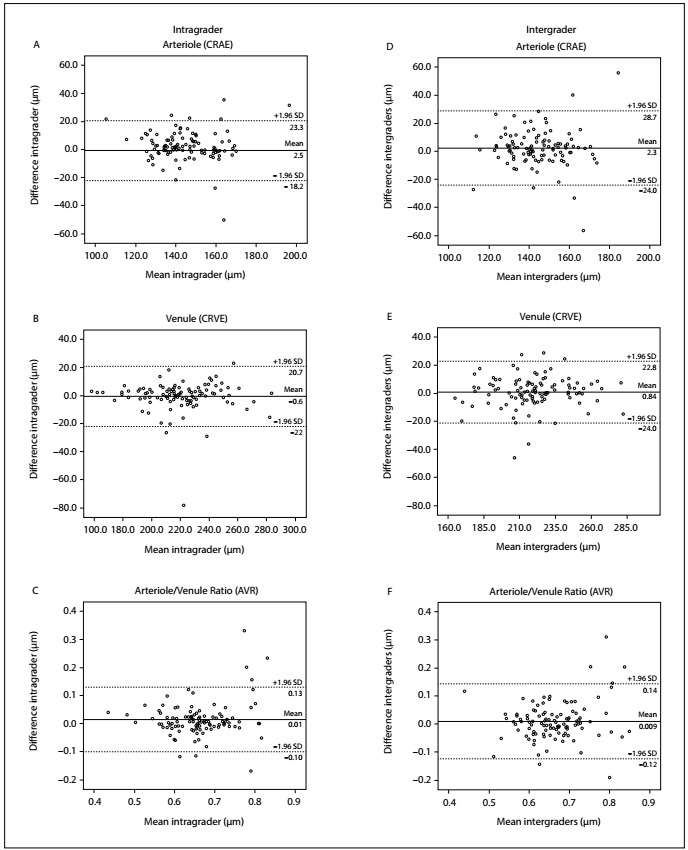
Reproducibility evaluation on Bland-Altman plots of the intra and intergrader differences in retinal microvascular measurements. Dashed lines indicate the 95% confidence interval (CI) of the mean difference, which is indicated by the black line.

Bland-Altman plots (Figure 2) showed that the differences obtained between the repeated readings in ELSA-Brasil were distributed in a similar way over the spectrum of values. In the intragrader measurements, the differences were < 10 µm in 75.2% of the arteriole measurements (CRAE) and in 86.7% of the venous measurements (CRVE). For the variable arteriole/venule ratio (AVR), 90.5% of the sample showed a difference < 0.1. In the intergrader measurements, the differences did not reach 10 µm in 71.4% of the arteriole measurements (CRAE) and 74.3% of the venule measurements (CRVE). For the variable arteriole/venule ratio (AVR), 89.5% of the sample showed a difference < 0.1.

## DISCUSSION

The validity of the retinal vessel diameter measurements in ELSA-Brasil, based on repeated measurements on a sample of 25 images received from the Department of Ophthalmology and Visual Sciences of the University of Wisconsin, was excellent, such that only trivial differences were found. The reproducibility of determinations of these diameters, characterized through repeated grading of 105 images from the ELSA-Brasil study that were chosen to present values across the spectrum of those obtained in the study, was high for the basic measurements and moderate for their ratio.

Regarding validity, the differences in comparison with the gold standard were on average less than 1% of the absolute values of the diameters, as can be seen by comparing mean differences (Y axis) with mean values (X axis) in the Bland-Altman graphs. These differences, besides being epidemiologically irrelevant, were not statistically significant.

In general, the reproducibility of measurements in epidemiological studies involves both biological variability and the variability of the entire data collection method. This can include the biological variability of the retina between repeated measurements, variability in capturing the images through photography and variability in the process of grading those images. Thus, a hypothetical overall ICC would represent the correlation between repeated retinal evaluations on an individual involving photography at different times, use of different cameras, assessment of the various images by different graders and repeated gradings by each grader. In this study, we only considered the reproducibility of grading. Regarding this reproducibility, the agreement between measurements performed repeatedly by the same evaluator was high for both arteriolar and venular diameter, with ICC > 0.77 and ICC > 0.90 respectively, and the concordance between observers was high, with ICC > 0.70 for both measurements. The intra and intergrader agreements for the AVR (ICCs of 0.70 and 0.68, respectively) were at the top of the “moderate” range of agreement.

These results are compatible with those of other studies described in the literature. In the Atherosclerosis Risk Communities Study (ARIC), the reproducibility of the readings was estimated as 140 retinal images for intragrader agreement and 151 images for intergrader agreement. The concordance was high for the venous diameter, with ICCs of 0.89 and 0.77 for intra and intergrader agreement, respectively.^1^ In the Beaver Dam Eye Study (BDES), reproducibility was evaluated in relation to 40 retinal images, with ICCs between 0.78 and 0.99 for inter and intragrader agreement,^25^ presumably considering all three microvascular measurements. The Cardiovascular Health Study (CHS) described intra and intergrader reproducibility in relation to 71 and 69 subjects, respectively, with ICCs of 0.67 and 0.91, but also without indicating the microvascular measurement of the retina to which these related.^26^ In the Singapore Cohort Study of Risk Factors for Myopia (SCORM), in relation to 50 images, the researchers evaluated intragrader reproducibility with only one evaluator. The ICC obtained for CRAE was 0.85 and for CRVE was 0.97, thus indicating high reproducibility for this grader.^27^ In the Singapore Malay Eye Study (SIMES), 44 images were evaluated and intragrader ICCs of 0.88 for CRAE and 0.92 for CRVE were estimated, and intergrader ICCs of 0.88 for CRAE and 0.87 for CRVE.^20^^,^^28^ Thus, we believe that our reproducibility results are consistent with those found in other studies, especially considering the relatively large time interval between measurements (about two years) and the larger number of readers who formed part of the team in ELSA-Brasil than were present in most of these other studies.

Retinal microvascular measurements are important for better comprehension of most chronic diseases. A growing number of published reports containing retinography data have expanded the range of metabolic and vascular diseases (cardiovascular diseases, diabetes mellitus, hypertension, stroke, obesity and dyslipidemia)^9^^,^^21^^,^^25^^,^^26^ that seem to involve microvascular alterations in association with their development and/or progression. Aside from these better studied associations, retinal microvascular measurements have more recently also been shown to be associated with cognitive dysfunction,^31^^,^^32^ the prevalence of complications in type 1 diabetes,^33^ the internal carotid artery pulsation index^34^ and changes to adiponectin levels.^35^

## CONCLUSION

In summary, our study suggests that the retinal microvascular measurements in the ELSA-Brasil have strong validity and moderate to high reproducibility within and across graders. The capacity for generalization of these findings to other studies is restricted to the use of the same techniques, software and training procedures. Future studies analyzing associations of these microvascular retinal measurements with clinical and subclinical measurements of disease in ELSA-Brasil may contribute towards better understanding of changes within the disease-health process and towards prediction of the onset of these diseases.

## Supplementary material

The statistical basis for measurement of the intraclass correlation coefficient when different numbers of measurements are read by each grader, but there is a constant number of readings per person, as developed by Dr. Lloyd E. Chambless, was used in this study. We applied the method of moments, as follows.

Y is the variable whose repeatability we study.

$$Y_{ijk}=\;\;\mu +\;\alpha_{i}+\;\beta_{j}+\;\epsilon_{ijk}$$

*α* ~ *N* (0, *σ* ^*2*^_*α*_ ),*β* ~ *N* (0, *σ* ^*2*^_*β*_ ), ∈ ~ *N* (0, *σ* ^*2*^_*e*_ )

People (α , ID) Reader (β, R) Repeats (∈, e)

i = 1, …,n j = 1, …p k = 1, …, *n*_*ij*_

For any ID =i, $m_{i=}\begin{array}{c} p\\ \sum \;\\ j=1 \end{array}n_{ij}$ for all *i*, so m_i_ = obs per *i*.

Assume m_i_=*m* for all ID. (Exclude observations for which this is false or keep only m if there are more).

We will work with the mean sums of squares:

$${MSS}_{\alpha }=\frac{1}{n-1}\left[\left(\begin{array}{c} n\\ \sum \;\\ i=1\; \end{array}\frac{{Y²}_{i..}}{m}\right)-\frac{{Y²}_{...}}{nm}\right]$$

$${MSS}_{\beta }=\;\frac{1}{p-1}\left[\left(\begin{array}{c} p\\ \sum \;\\ j=1\; \end{array}\frac{{Y²}_{.j.}}{n_{j}}\right)-\frac{{Y²}_{...}}{nm}\right]$$

$${MSS}_{e}=\frac{1}{g}\left[\left(\begin{array}{c} n\\ \sum \;\\ i=1\; \end{array}\begin{array}{c} p\\ \sum \;\\ j=1 \end{array}\;\;I(n_{ij}\geq 2)\;\left(\begin{array}{c} n_{ij}\\ \sum \;\\ k=1\;\; \end{array}{Y^{2}}_{ijk}\;\;-\;\frac{{Y²}_{ij.}}{n_{ij}}\right)\right)\right]$$

where

$n_{j}\;=\;\;\begin{array}{c} n\\ \sum \\ i=1\; \end{array}n_{ij}$, *I*(*n*_*ij*_ ≥ 2) is 1 when *n*_*ij*_ ≥ 2 and 0 otherwise, and g=n∑ i=1 p∑ j=1Inij≥2(nij-1).

$$Y_{i..}=\;\begin{array}{c} p\\ \sum \\ j=1 \end{array}\begin{array}{c} n_{ij}\\ \sum \\ k=1\;\; \end{array}Y_{ijk}=m\left(µ+\;\alpha_{i}\right)+\;\begin{array}{c} p\\ \sum \\ j=1 \end{array}\begin{array}{c} n_{ij}\\ \sum \\ k=1\;\; \end{array}\beta_{j\;\;}+\;\begin{array}{c} p\\ \sum \\ j=1 \end{array}\begin{array}{c} n_{ij}\\ \sum \;\\ k=1\;\; \end{array}\epsilon_{ijk}$$

$$=m\left(µ+\;\alpha_{i}\right)+\;\begin{array}{c} p\\ \sum \\ j=1 \end{array}n_{ij}\beta_{j\;\;}+\;\begin{array}{c} p\\ \sum \\ j=1 \end{array}\begin{array}{c} n_{ij}\\ \sum \;\\ k=1\;\; \end{array}\epsilon_{ijk}$$

$$Y_{\ldots }=\;\begin{array}{c} n\\ \sum \\ i=1 \end{array}Y_{i}..\;=\;\;m\;n\;µ+m\;\alpha_{.}+\;\begin{array}{c} n\\ \sum \;\\ i=1\; \end{array}\begin{array}{c} p\\ \sum \;\\ j=1\; \end{array}n_{ij}\beta_{j}\;+\begin{array}{c} n\\ \sum \\ i=1 \end{array}\begin{array}{c} p\\ \sum \\ j=1 \end{array}\begin{array}{c} n_{ij}\\ \sum \\ k=1\;\; \end{array}\epsilon_{ijk}$$

$$=\;\;m\;n\;µ+m\;\alpha_{.}+\;\begin{array}{c} p\\ \sum \;\\ j=1\; \end{array}(\begin{array}{c} n\\ \sum \;\\ i=1\; \end{array}n_{ij})\;\beta_{j}\;+\sum_{i\;}\sum_{j\;}\sum_{k}\epsilon_{ijk}$$

for $\alpha_{.}=\;\;\begin{array}{c} n\\ \sum \\ i=1 \end{array}\alpha_{i}\;$.

Note that $\left(\begin{array}{c} n_{ij}\\ \sum \;\\ k=1\;\; \end{array}{Y^{2}}_{ijk}\;\;-\;\frac{{Y²}_{ij.}}{n_{ij}}\right)=0$ when *n*_*ij*_ = 1.

$$E\;\left({MSS}_{\alpha }\right)=\;\frac{1}{n-1}\left[\left(\begin{array}{c} n\\ \sum \\ i=1\; \end{array}\frac{E\left({Y^{2}}_{i..}\right)}{m}\right)-\frac{E\left({Y^{2}}_{\ldots }\right)}{nm}\right]$$

$${Y²}_{i..}={\left(m\mu \right)}^{2}+\;\begin{array}{c} p\\ \sum \\ j=1 \end{array}{n_{ij}}^{2}\beta_{j}^{2}\;+\;m^{2}{\alpha_{i}}^{2}+\begin{array}{c} p\\ \sum \\ j=1 \end{array}\begin{array}{c} n_{ij}\\ \sum \\ k=1 \end{array}{\epsilon ²}_{ijk}+\;\;cross\;product\;terms\;with\;zero\;expectation.$$

$$E\;({Y²}_{i..})\;={\left(m\;\mu \right)}^{2}+\left(\begin{array}{c} p\\ \sum \\ j=1 \end{array}{n²}_{ij}{\sigma^{2}}_{\beta }\right)+\;m^{2}{\sigma^{2}}_{\alpha }\;+\;\;m\;{\sigma^{2}}_{e}$$

$${Y^{2}\ldots =m^{2}n^{2}{µ}^{2}+\;\;m^{2}\alpha ²}_{.}\;+\;\;\begin{array}{c} p\\ \sum \\ j=1 \end{array}{\left(\begin{array}{c} n\\ \sum \\ i=1\; \end{array}n_{ij}\right)}^{2}{\beta ²}_{j}\;+\;\begin{array}{c} n\\ \sum \;\\ i=1\; \end{array}\begin{array}{c} p\\ \sum \\ j=1\; \end{array}\begin{array}{c} n_{ij}\\ \sum \\ k=1 \end{array}{\epsilon ²}_{ijk}\;+terms\;with\;zero\;expectation$$

$$E\;\left(Y^{2}\ldots \right)=\;m^{2}n^{2}{µ}^{2}+\;\;m^{2}\;n\;{\sigma ²}_{\alpha }\;+\begin{array}{c} p\\ \sum \\ j=1\; \end{array}{\left(\begin{array}{c} n\\ \sum \\ i=1\; \end{array}n_{ij}\right)}^{2}{\sigma ²}_{\beta }+n\;m\;{\sigma ²}_{e}$$

$$E\;\left({MSS}_{\alpha }\right)=\;\frac{1}{m\left(n-1\right)}\left[nm²\mu ²+\left(\begin{array}{c} n\\ \sum \\ i=1 \end{array}\begin{array}{c} p\\ \sum \\ j=1 \end{array}\;\;nij^{2}\right){\sigma^{2}}_{\beta }\;+n\;m^{2}{\sigma^{2}}_{\alpha }+\;n\;m\;{\sigma^{2}}_{e}\;-\;\left(m^{2}n\;{µ}^{2}+\;\;m^{2}{\sigma^{2}}_{\alpha }+\;\;\frac{1}{n}\begin{array}{c} p\\ \sum \\ j=1\; \end{array}{\left(\begin{array}{c} n\\ \sum \\ i=1\; \end{array}n_{ij}\right)}^{2}{\sigma^{2}}_{\beta }+\;m\;{\sigma^{2}}_{e}\right)\right]$$

$$=\;\frac{1}{m\left(n-1\right)}\left[\left(n-1\right)m^{2}{\sigma^{2}}_{\alpha }+\;\;\left(\begin{array}{c} n\\ \sum \\ i=1\; \end{array}\begin{array}{c} p\\ \sum \\ j=1\; \end{array}{n^{2}}_{ij}\;-\;\frac{1}{n}\begin{array}{c} p\\ \sum \\ j=1\; \end{array}{\left(\begin{array}{c} n\\ \sum \\ i=1\; \end{array}n_{ij}\right)}^{2}\right){\sigma^{2}}_{\beta }+\left(n-1\right)m\;{\sigma^{2}}_{e}\right]$$

$$=m\;{\sigma^{2}}_{\alpha }+\;\frac{1}{m\left(n-1\right)}\left(\begin{array}{c} n\\ \sum \\ i=1\; \end{array}\begin{array}{c} p\\ \sum \\ j=1\; \end{array}{n^{2}}_{ij}\;-\;\frac{1}{n}\begin{array}{c} p\\ \sum \\ j=1\; \end{array}{\left(\begin{array}{c} n\\ \sum \\ i=1\; \end{array}n_{ij}\right)}^{2}\right){\sigma ²}_{\beta \;\;}+\;{\sigma ²}_{e\;}$$

$$E\left({MSS}_{\alpha }\right)=m{\sigma^{2}}_{\alpha }+\;\frac{1}{m\left(n-1\right)}\left(\begin{array}{c} n\\ \sum \\ i=1\; \end{array}\begin{array}{c} p\\ \sum \\ j=1\; \end{array}{n^{2}}_{ij}-\;\;\frac{1}{n}\begin{array}{c} p\\ \sum \\ j=1\; \end{array}{n^{2}}_{j}\right){\sigma ²}_{\beta }+\;{\sigma ²}_{e}$$
(A)

Where $n_{j}\;=\;\;\begin{array}{c} n\\ \sum \\ i=1\; \end{array}n_{ij}$

$$Y_{.j.}=\;\begin{array}{c} n\\ \sum \\ i=1\; \end{array}\begin{array}{c} n_{ij}\\ \sum \\ k=1\; \end{array}Y_{ijk}=\;\;\;\begin{array}{c} n\\ \sum \\ i=1\; \end{array}\begin{array}{c} n_{ij}\\ \sum \\ k=1\; \end{array}\left(\;\mu +\;\alpha_{i}+\;\beta_{j}\;+\epsilon_{ijk}\right)$$

$$=\;n_{j}µ+\begin{array}{c} n\\ \sum \\ i=1\; \end{array}n_{ij}\alpha_{i}\;+\;\;n_{j}\beta_{j}\;+\;\;\begin{array}{c} n\\ \sum \\ i=1\; \end{array}\begin{array}{c} n_{ij}\\ \sum \\ k=1\; \end{array}\epsilon_{ijk}$$

$${(Y}_{.j.})²=\;{n^{2}}_{j}\;µ²\;+\;\begin{array}{c} n\\ \sum \\ i=1\; \end{array}{n^{2}}_{ij}{\alpha^{2}}_{i}+\;{n^{2}}_{j}{\beta^{2}}_{j\;}\;+\;\;\begin{array}{c} n\\ \sum \\ i=1\; \end{array}\begin{array}{c} n_{ij}\\ \sum \\ k=1\; \end{array}{\epsilon ²}_{ijk}\;\;+cross\;product\;terms\;with\;zero\;expected\;value$$

$$E\left({(Y}_{.j.})²\;\right)=\;{n^{2}}_{j}{µ}^{2}+\left(\begin{array}{c} n\\ \sum \\ i=1\; \end{array}{n^{2}}_{ij}\right){\sigma^{2}}_{\alpha }+\;\;{n^{2}}_{j}{\sigma^{2}}_{\beta \;}\;+\;\;n_{j}{\sigma ²}_{e}$$

$$E\left({MSS}_{\beta }\right)=\;\;\frac{1}{p-1}\left[\left(\begin{array}{c} p\\ \sum \\ j=1\; \end{array}\frac{E\left(Y.j^{2}.\right)}{n_{j}}\right)-\;\frac{E\;\left(Y^{2}\ldots \right)}{nm}\right]$$

$$=\;\frac{1}{p-1}\left[\begin{array}{c} p\\ \sum \\ j=1\; \end{array}\left(n_{j}{µ}^{2}+\;\frac{\left(\begin{array}{c} n\\ \sum \\ i=1\; \end{array}{n^{2}}_{ij}\right)}{n_{j}}{\sigma^{2}}_{\alpha }+\;n_{j}{\sigma^{2}}_{\beta \;}+\;\;\;{\sigma^{2}}_{e}\right)-\;\left(\;m\;n\;{µ}^{2}+\;\;m\;{\sigma^{2}}_{\alpha }\;+\;\;\begin{array}{c} p\\ \sum \\ j=1\; \end{array}{n^{2}}_{j}\frac{{\sigma^{2}}_{\beta \;}}{mn}+\;{\sigma^{2}}_{e}\right)\right]$$

$$=\;\frac{1}{p-1}\left[\left(\begin{array}{c} p\\ \sum \\ j=1\; \end{array}n_{ij}\right){µ}^{2}\;+\begin{array}{c} p\\ \sum \\ j=1\; \end{array}\frac{\left(\begin{array}{c} n\\ \sum \\ i=1\; \end{array}{n^{2}}_{ij}\right)}{n_{j}}{\sigma^{2}}_{\alpha }+\frac{\left(\begin{array}{c} p\\ \sum \\ j=1\; \end{array}n_{j}\right)}{mn}{\sigma^{2}}_{\beta \;}+p{{\;\delta }^{2}}_{e}-\;\left(\;m\;n\;{µ}^{2}+\;m\;{\sigma^{2}}_{\alpha }+\;\frac{\begin{array}{c} p\\ \sum \\ j=1\; \end{array}{n^{2}}_{j}}{m\;n}{\sigma^{2}}_{\beta \;}+\;{\sigma^{2}}_{e}\right)\right]$$

$$E\;\left({MSS}_{\beta }\right)=\;\frac{1}{p-1}\left[\left(\begin{array}{c} p\\ \sum \\ j=1\; \end{array}\frac{\left(\begin{array}{c} n\\ \sum \\ i=1\; \end{array}{n^{2}}_{ij}\right)}{n_{j}}-m\right){\sigma^{2}}_{\alpha }+\;\frac{1}{p-1}\left(n\;m-\;\frac{\begin{array}{c} p\\ \sum \\ j=1\; \end{array}{n^{2}}_{j}}{m\;n}\right){\sigma^{2}}_{\beta \;}+\left(p-1\right){{\;\sigma }^{2}}_{e}\right]$$

$$E\;\left({MSS}_{\beta }\right)=\;\frac{1}{\left(p-1\right)}\left(\begin{array}{c} p\\ \sum \\ j=1\; \end{array}\frac{\left(\begin{array}{c} n\\ \sum \\ i=1\; \end{array}{n^{2}}_{ij}\right)}{n_{j}}-m\right){\sigma^{2}}_{\alpha }+\;\frac{1}{\left(p-1\right)}\left(mn-\;\frac{\begin{array}{c} p\\ \sum \\ j=1\; \end{array}{n^{2}}_{j}}{mn}\right){\sigma^{2}}_{\beta }+\;\;{\sigma^{2}}_{e}$$
(B)

Suppose *n*_*ij*_ = w for all i, j. Then $m=\;\begin{array}{c} p\\ \sum \\ j=1\; \end{array}n_{ij}=pw=OBS$*per person*, $u=\;n_{j}=\;\begin{array}{c} n\\ \sum \\ i=1\; \end{array}n_{ij}=nw=OBS$ per reader, and mn = pwn = pu.

The coefficient of σ^2^_β_ in the *E* (*MSS*_*β*_ ) formula simplifies as follows:

$$\frac{1}{\left(p-1\right)}\left(m\;n\;\;\frac{\left(\begin{array}{c} p\\ \sum \\ j=1\; \end{array}{n^{2}}_{j}\right)}{mn}\right)=\;\frac{1}{\left(p-1\right)}\left(\;pu\;-\;\frac{pu^{2}}{pu}\right)$$

$$=\;\frac{1}{\left(p-1\right)}\left(\;pu\;-\;u\;\right)=\;\frac{1}{\left(p-1\right)}\left(p-1\right)\;u=\;\;u$$

What is the coefficient of σ^2^_β_ in the *E* (*MSS*_*α*_ ) formula if *n*_*ij*_ = *w* for all *i*, *j*?

u = OBS per reader = nw, w = OBS per reader x ID, m = OBS per person = pw

$$\frac{1}{m\;(n-1)}\left(\begin{array}{c} n\\ \sum \\ i=1\; \end{array}\begin{array}{c} p\\ \sum \\ j=1\; \end{array}w^{2}-\;\frac{\;1\;}{\;n\;}\begin{array}{c} p\\ \sum \\ j=1\; \end{array}u^{2}\right)=\;\frac{1}{m\;(n-1)}\left(\;p\;n\;w^{2}-\;\frac{\;1\;}{\;n\;}\;pu^{2}\right)$$

$$=\;\frac{1}{pw\;\left(n-1\right)}\left(p\;u\;w-\;\frac{\;1\;}{\;n\;}\;pu^{2}\right)$$

$$=\;\frac{1}{pw\;\left(n-1\right)}\;p\;u\;\left(w-\;\;\frac{\;u\;}{\;n\;}\right)=\;\;\left(w-\;\frac{nw}{w}\right)=0$$

What is the coefficient of σ^2^_α_ in the *E* (*MSS*_*β*_ ) formula *n*_*ij*_ = *w* for all *i*, *j*?

$$\frac{1}{p-1}\left(\begin{array}{c} p\\ \sum \\ j=1\; \end{array}\frac{\left(\begin{array}{c} n\\ \sum \\ i=1\; \end{array}{n^{2}}_{ij}\right)}{n_{j}}-m\right)=\;\frac{1}{\left(p-1\right)}\left(\begin{array}{c} p\\ \sum \\ j=1\; \end{array}\frac{\left(\begin{array}{c} n\\ \sum \\ i=1\; \end{array}w^{2}\right)}{nw}-pw\right)$$

$$=\;\frac{1}{p-1}\left(\begin{array}{c} p\\ \sum \\ j=1\; \end{array}\frac{nw^{2}}{nw}-pw\;\right)=\;\frac{1}{p-1}\left(\begin{array}{c} p\\ \sum \\ j=1\; \end{array}w\;-\;\;pw\right)$$

$$=\;\frac{1}{p-1}\left(pw-pw\right)=0\;!$$

Thus, if *n*_*ij*_ = *w* for all *i*, *j* then the formula for *E* (*MSS*_*β*_ ) and *E* (*MSS*_*α*_ ) simplifies to what SAS procglm would get.

We return to the other expected squares.

$${Y²}_{ijk}=\;{µ}^{2}+\;\;{\alpha ²}_{i}\;+\;{\beta ²}_{j}\;+\;\;{\epsilon ²}_{ijk}+\;\;cross\;product\;terms\;with\;zero\;expectation.$$

$$E\left({Y^{2}}_{ijk}\right)=\;{µ}^{2}+\;\;{\sigma ²}_{\alpha }\;+\;{\sigma ²}_{\beta }+\;\;{\sigma ²}_{e}$$

$$Y_{ij.\;}\;=\;\;\begin{array}{c} n_{ij}\\ \sum \\ k=1\; \end{array}Y_{ijk}=\;\;\begin{array}{c} n_{ij}\\ \sum \\ k=1\; \end{array}\left(\;µ+\;\alpha_{i}\;+\;\;\beta_{j}\;+\;\epsilon_{ijk}\right)$$

$${=\;n}_{ij}\mu +\;n_{ij}\alpha_{i}+\;\;\;n_{ij}\beta_{j}\;+\begin{array}{c} n_{ij}\\ \sum \\ k=1\; \end{array}\epsilon_{ijk}$$

$${Y^{2}}_{ij.}=\;{n^{2}}_{ij}{µ}^{2}+\;\;{n^{2}}_{ij}{\alpha ²}_{i}+\;{n^{2}}_{ij}{\beta ²}_{j}+\begin{array}{c} n_{ij}\\ \sum \\ k=1\; \end{array}{\epsilon ²}_{ijk}+\;cross\;product\;terms\;with\;zero\;expectation$$

$$E\left({Y^{2}}_{ij.}\right)=({n^{2}}_{ij}\mu^{2}+\;{n^{2}}_{ij}{\sigma^{2}}_{\alpha }+\;{n^{2}}_{ij}{\sigma^{2}}_{\beta }+\;n_{ij}{\sigma ²}_{e}$$

With *I*(*n*_*ij*_ ≥ 2) = 1 when *n*_*ij*_ ≥ 2 and 0 otherwise,

$${E(MSS}_{e})=\;\frac{1}{g}\begin{array}{c} n\\ \sum \\ i=1\; \end{array}\begin{array}{c} p\\ \sum \\ j=1\; \end{array}I\left(n_{ij}\geq 2\right)\left(\begin{array}{c} n_{ij}\\ \sum \\ k=1\; \end{array}\left(\;E({Y^{2}}_{ijk}\right)-\;\;\frac{E\;\left({Y^{2}}_{ij.}\right)}{n_{ij}}\right)$$

$$=\frac{1}{g}\begin{array}{c} n\\ \sum \\ i=1\; \end{array}\begin{array}{c} p\\ \sum \\ j=1\; \end{array}I\left(n_{ij}\geq 2\right)\;(\begin{array}{c} n_{ij}\\ \;\sum \\ k=1\; \end{array}\left({µ}^{2}+\;{\sigma^{2}}_{\alpha }+\;{\sigma^{2}}_{\beta }+\;{\sigma^{2}}_{e}\right)\;{-\;(n}_{ij}\mu^{2}+\;n_{ij}{\sigma^{2}}_{\alpha }+\;\;\;n_{ij}{\sigma^{2}}_{\beta }\;+\;n_{ij}{\sigma^{2}}_{e}))$$

$$\frac{1}{g}\begin{array}{c} n\\ \sum \\ i=1\; \end{array}\begin{array}{c} p\\ \sum \\ j=1\; \end{array}I\;I\;\left(n_{ij}\geq 2\right)$$

$$\left(\left(n_{ij}\mu^{2}+\;n_{ij}{\sigma^{2}}_{\alpha }+\;n_{ij}{\sigma^{2}}_{\beta }\;+\;n_{ij}{\sigma^{2}}_{e}\right)-\left(n_{ij}\mu^{2}+\;n_{ij}{\sigma^{2}}_{\alpha }+\;\;\;n_{ij}{\sigma^{2}}_{\beta }\;+\;{\sigma^{2}}_{e}\right)\right)$$

$=\frac{1}{g}\begin{array}{c} n\\ \sum \\ i=1\; \end{array}\begin{array}{c} p\\ \sum \\ j=1\; \end{array}\;I\;\left(n_{ij}\geq 2\right)\left(n_{ij}-1\right){\sigma^{2}}_{e}=\;{\sigma^{2}}_{e}$, since

$$g=\begin{array}{c} n\\ \sum \;\\ i=1\; \end{array}\begin{array}{c} p\\ \sum \;\\ j=1 \end{array}I\left(n_{ij}\geq 2\right)(n_{ij}-1)$$

$$Thus,\;{E(MSS}_{e})=\;{\sigma^{2}}_{e}.$$
(C)

We use equations (A), (B), and (C) above to derive estimates for σ^2^_α_, σ^2^_β_, and σ^2^_e_, replacing the expected mean sums of squares by the observed values, and solving the equations.

From (C),

σ^²e =1gn∑i=1 p∑j=1  I nij-2nij∑k=1 Y2ijk- Y2ij.nij

From (B),

1(p-1)p∑j=1 n∑i=1 n2ijnj-mσ^2α+ 1(p-1)m n- p∑j=1 n2jm nσ^2β =  MSSβ- σ^²e
(D)

From (A),

mσ^2α+ 1mn-1n∑i=1 p∑j=1 n2ij- 1np∑j=1 n2jσ^²β=MSSα- σ^2e
(E)

Thus, with (D) and (E) we have two equations in two unknowns σˆ^2^_*α*_ and σˆ^2^_*β*_ . We have to solve this two equations for the unknown variances.

Then,  σ^²total= σ^²α+  σ^²β+  σ^²e , for σˆ^2^_*α*_ the between-person variance, σˆ^2^_*β*_ the between-reader variance, and σˆ^2^_*e*_ the error variance, which combines within-reader and within-person variability.

The ICC:Corr(Yijk, Yij’k’) =σ^²α / σ^²total     for j≠j´ (different readers)

CorrYijk, Yijk’=(σ^²α +  σ^²β)  / σ^²total      for k≠k´ (same reader)
